# Discovery of novel natural products for mosquito control

**DOI:** 10.1186/s13071-022-05594-z

**Published:** 2022-12-21

**Authors:** Cecilia S. Engdahl, Chinmay V. Tikhe, George Dimopoulos

**Affiliations:** 1 W. Harry Feinstone Department of Molecular Microbiology and Immunology, Bloomberg School of Public Health, Johns Hopkins University, Baltimore, MD USA; 2 Present Address: Department of Clinical Microbiology, Virology, Umeå University, 90185 Umeå, Sweden

**Keywords:** Vector control, Biopesticide, Natural products, Mosquito-borne diseases, *Aedes aegypti*, *Anopheles gambiae*

## Abstract

Vector control plays a key role in reducing the public health burden of mosquito-borne diseases. Today’s vector control strategies largely rely on synthetic insecticides that can have a negative environmental impact when applied outdoors and often become inefficient because of the mosquitoes’ ability to develop resistance. An alternative and promising approach to circumvent these challenges involves the implementation of insecticides derived from nature (biopesticides) for vector control. Biopesticides can constitute naturally occurring organisms or substances derived from them that have lifespan-shortening effects on disease vectors such as mosquitoes. Here we present the discovery and evaluation of natural product-based biological control agents that can potentially be developed into biopesticides for mosquito control. We screened a natural product collection comprising 390 compounds and initially identified 26 molecules with potential ability to kill the larval stages of the yellow fever mosquito *Aedes aegypti*, which is responsible for transmitting viruses such as dengue, Zika, chikungunya and yellow fever. Natural products identified as hits in the screen were further evaluated for their suitability for biopesticide development. We show that a selection of the natural product top hits, bactobolin, maytansine and ossamycin, also killed the larval stages of the malaria-transmitting mosquito *Anopheles gambiae* as well as the adult form of both species. We have further explored the usefulness of crude extracts and preparations from two of the best candidates’ sources (organisms of origin) for mosquitocidal activity, that is extracts from the two bacteria *Burkholderia thailandensis* and *Streptomyces hygroscopicus* var. *ossamyceticus*.

**Graphical abstract:**

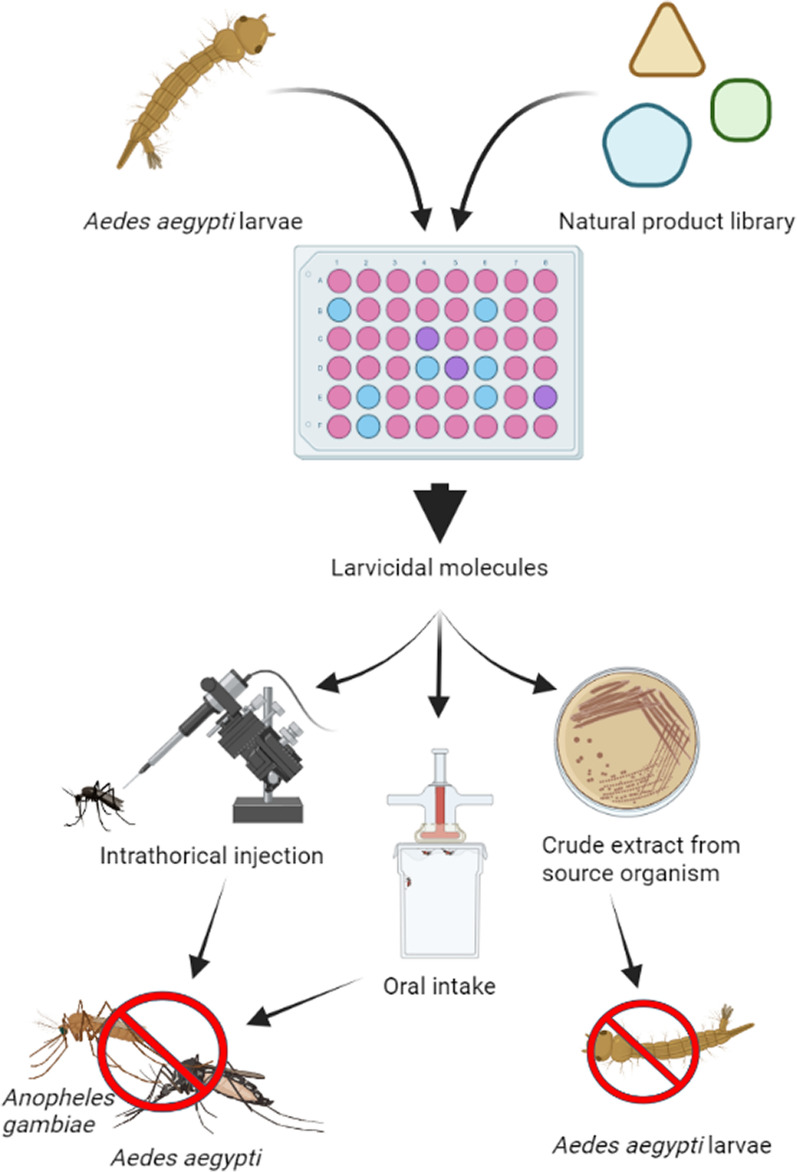

**Supplementary Information:**

The online version contains supplementary material available at 10.1186/s13071-022-05594-z.

## Background

Vector-borne infections continue to be a major public health concern; approximately 80% of the world’s population is at risk of acquiring a vector-borne disease [1]. Malaria, dengue, Zika, chikungunya, yellow fever and other mosquito-borne diseases cause more than 1 million deaths each year worldwide and are responsible for a tremendous socio-economic burden, primarily in underdeveloped regions [2–6].

Efficient vector control is the most effective preventive strategy, and the encouraging reports of a decline in the number of malaria cases in the beginning of the twenty-first century was primarily attributed to the use of insecticide-based vector control [7]. Estimations in the latest World Malaria report (2021) are that 1.7 billion cases and 10.6 million deaths were averted between 2000 and 2020 [2].

However, vector control relies primarily on chemical insecticides with very few targets, and these insecticides have recently been increasingly associated with greater resistance, off-target toxicity and long-lasting persistence in nature [8]. Natural products (NP), commonly secondary metabolites produced by bacteria, plants or fungi, are considered a promising alternative to synthetic insecticidal compounds with respect to these concerns [8, 9]. The generally lesser toxicity and environmental effects of biopesticides have been addressed in the agricultural sector where the need for alternative methods, and thus the interest in biopesticides, has greatly increased [10, 11]. Historically, extracts and compounds isolated from natural resources have contributed to many aspects of medicinal health through their antibiotic, antitumor, antifungal, antibacterial or antiparasitic activity [12, 13].

The most well-established NP-based biopesticide used for mosquito-control today consists of crystals of *Bacillus thuringiensis* var. *israelensis* (Bti) [14]. Its benefits are linked to its mosquito-specific mode of action and the fact that no resistance has been reported from the field despite its use for > 30 years [15]. The main drawback is the timing and the need for repeated distribution during the season, and another limitation is that the products available to date are only used for targeting the larval stage of the mosquito life cycle. Natural sources that are currently being explored for the development of new mosquitocidal activities include fungi [16, 17], plants [18–25] and microbes [26–31], with plants being the most highly represented.

Despite a resurgence in investigating natural sources for new products with mosquitocidal activity, very few NPs have made it to the finish line and become approved products for vector control [32]. The reason for this failing is usually one or more challenges related to toxicity, stringent regulatory requirements, potency, costs, access and/or supply. Also, from a marketing standpoint, a new product must be demonstrated to be more advantageous than already marketed products [33]. In the search for new targets to circumvent problems with rapid resistance development and off-target toxicity, one approach is to re-purpose natural products already investigated and characterized for other purposes [34]. We reasoned that screening compound collections with well-documented toxicity and physico-chemical properties could accelerate the product development pipeline and avoid some of the common challenges related to NP discovery.

Here we have aimed at discovering new mosquitocidal NPs from a library of isolated, already known naturally derived compounds, thereby having access to knowledge about potential hits at the initial screening stage. Based on the promising results from our initial screen, in which > 25 NP compounds displayed larvicidal activity against the yellow fever mosquito *Aedes aegypti*, we then further evaluated a selection of the candidate NPs. Several of these proved active also against adult *Aedes* mosquitoes as well as against the major malaria mosquito vector *Anopheles gambiae* via several different delivery/exposure routes. In the concluding phase of our study, we went back to the bacterial source of origin of the most promising NP hit compounds bactobolin and ossamycin and verified that a crude extract of the secondary metabolites produced by these bacteria retained mosquitocidal activity. These two NPs stood out from the other hits as they were potent against both larvae and adult life stages. Our results suggest that this underexplored approach is an efficient method for searching for new mosquito-control products and encourages a deeper exploration of the ribosome inhibitor bactobolin from *Burkholderia thailandensis* and the ATPase inhibitor ossamycin from *Streptomyces hygroscopicus* var. *ossamyceticus* as potential biopesticide products.

## Methods

### Mosquito strains and rearing

Two mosquito species were used in this study: the yellow fever mosquito *Ae. aegypti*, Rockefeller strain [35], and the malaria mosquito *An. gambiae*, Keele strain [36]. Mosquitoes were reared under standard optimal conditions at 27 °C and 80% relative humidity on a 12 h light/dark cycle. Larvae were maintained at a density of approximately 100 larvae/l deionized water from the first instar onward and fed with ground tropical fish flakes (TetraMin) and, for *An. gambiae* only, cat food pellets (Purina Cat Chow). Adults were kept in 25 × 25 × 25-cm metal cages and provided a 10% sucrose solution. For experiments in which a smaller and defined number of females were used, the mosquitoes were kept in 235-ml cups (Solo Foodservice) covered by a mesh, and sucrose was provided via soaked cotton balls placed on top of the mesh.

### Screening for larvicidal natural products

A collection of 390 previously characterized and isolated NP compounds was tested for killing activity on *Ae. aegypti* larvae. The NP collection (Natural Products Set V) was a sub-selection from the Development Therapeutics Program (DTP) of the Open Repository Program collection at the National Cancer Institute (NCI), Bethesda, Maryland [37]. Factors in the selection were origin, structural diversity and purity (> 90% by ELSD, major peak has the correct mass ion). The NPs were provided and stored dissolved in DMSO at – 20 °C until use. The screen for larvicidal activity was performed on *Ae. aegypti* Rockefeller strain L2 larvae. Four larvae were placed in 1 ml hatching broth (1 l deionized water mixed with one tablet of finely ground tropical fish flakes, autoclaved) in a 48-well plate (Corning Falcon polystyrene microplates). Five microliters of the dissolved compound to be tested was added to each well, resulting in a final concentration of 50 µM compound and 0.5% DMSO. All NPs were tested once. There were six negative control wells (0.5% DMSO, no NP) per plate, and all samples were analyzed in relation to the controls on the same plate. The survival of the larvae was noted daily for 7 days.

### Establishing a threshold and confirming larvicidal activity

Any NPs identified as active in the screen that had previously been recorded as a reproductive toxin, carcinogenic, highly toxic, toxic or poisonous, was removed from consideration. In the screen, the NPs were tested only once, at a single concentration, and thus the hits were confirmed in the following study. The confirmation study was a replica of the initial screen, described in “Screening for larvicidal natural products” section, except that the larvae were also exposed to two additional, lower concentrations, 5 µM and 0.5 µM.

### Exploration of adulticidal activity

To identify a NP that possesses both larvicidal and adulticidal activity is highly desirable, and thus our next step was to investigate whether the confirmed hits also killed adult mosquitoes.

#### Microinjections

Thirty female mosquitoes, 5 to 7 days old, were injected with each NP at 20 or 2 mM using a Nanoject II Injector (Drummond). Females were knocked down by cold temperature and kept on an ice/cooling box during the injections. A volume of 69 nl of dissolved compound in mosquito saline (4 mM HEPES pH 6.9, 154 mM NaCl, 1.4 mM CaCl, 2.7 mM KCl) was injected into the thorax, and then the mosquitoes were left to wake up in a cup at room temperature. They were provided a 10% sucrose solution and thereafter kept under normal conditions. The % survival of the injected mosquitoes was noted at 24 and 48 h after injection. Thirty control mosquitoes were injected with the same volume of saline for each experiment (negative control). As a positive control (only in *Aedes* experiments) the commercially used carbamate insecticide propoxur was injected with the same two doses as the NPs plus one tenfold lower dose. The experiment was done in duplicate.

#### Feeding

Fifteen female mosquitoes, 3 to 4 days old, were starved for 3 to 4 h before being provided with a cotton ball soaked in a sucrose solution (10%) containing a NP (4 mM) in a final concentration of 4% DMSO. As controls (C), the mosquitoes were offered the sucrose solution with only DMSO (4%) or without any additives (blank). Propoxur (4 mM) was used as positive control. After 24 h the spiked sucrose was exchanged for a 10% sucrose solution, and survival of mosquitoes was monitored for 6 to 7 days. The experiment was done in triplicate.

### Bacterial culturing and secondary metabolite production

The sources (organisms of origin) of ossamycin [*Streptomyces hygroscopicus* var. *ossamyceticus* (ATCC-15420)] and bactobolin, [*Burkholderia thailandensis* (ATCC-700388)] were available and could be purchased from ATCC. Bacterial species identification was confirmed by ribosomal 16S gene amplification and sequencing followed by BLAST nucleotide searches against the NCBI database. For production of ossamycin, *S. hygroscopicus* was cultured in 2 ml of 3% tryptic soy broth, 10.3% sucrose and 0.5% yeast extract (TSBY) at 30 °C and 200 rpm for 3 days. Then, 1 ml was inoculated into 100 ml cultures of TSBY and grown for 7 days. For production of bactobolin, *B. thailandensis* was cultured in Luria-Bertani (LB) broth at 30 °C at 200 rpm. The LB broth (100 ml) was inoculated with 1 ml of an overnight culture and grown for 48 h. Thereafter, 5 ml of that culture was added to 350 ml of fresh LB broth and grown for an additional 48 h. All bacterial cultures were harvested by centrifugation at 7000 rpm for 12 min at 4 °C. The supernatants were filtered through a 0.22-µm filter, and the filtrates were stored at 4 °C until use.

Culturing and extraction of ossamycin and bactobolin were performed as described before, with modifications [38, 39]: a 1/10 volume of ethyl acetate was added to the filtered cultures and slowly shaken at 30 °C (80 rpm) for ~ 2 to 3 h. Samples were thereafter separated using a separatory funnel. The H_2_O phase (at the bottom) was mixed twice with ethyl acetate and shaken vigorously before being phase-separated. All three aliquots of the organic phase were pooled, and the liquid was evaporated in a Rotavapor (BÜCHI Labortechnik) using a water bath set at 60 °C. The remaining material in the round-bottom flask was dissolved in 1 ml (for the 100-ml cultures of *S. hygroscopicus*) or 2 ml (for the 300-ml cultures of *B. thailandensis*) of DMSO. The samples were stored at 4 °C until use.

To ensure that any possible mosquitocidal effect observed from the crude extracts was actually generated by extraction of metabolites and was not an effect of the growth medium or the extraction solvents, both the culturing and extraction were done in parallel on the same volume of un-inoculated culturing broth (no bacteria). These control samples were produced following the same protocol and dissolved in the same volume of DMSO as the bacterial samples and used as controls in the subsequent experiments.

### Evaluation of the larvicidal effect of crude extracts

Extractions from *S. hygroscopicus* and *B. thailandensis* bacterial cultures were tested for larvicidal activity on *Ae. aegypti* L2 larvae by addition to the larval breeding water. Volumes of 1, 10 or 100 µl of the crude extracts were added to 5 ml hatching broth in six-well plates, resulting in doses of 0.2, 2 and 20 µl/ml. Ten larvae were added per well, and each treatment was tested in triplicate. Larval survival was scored daily, and the data in the graphs represent the average survival of three replicates. Three control treatments were done: one with the addition of extracts of non-bacteria-containing culture medium, one with the addition of only DMSO and one with no addition.

## Results

### Initial screening of natural products for larvicidal activity

**Fig. 1:**
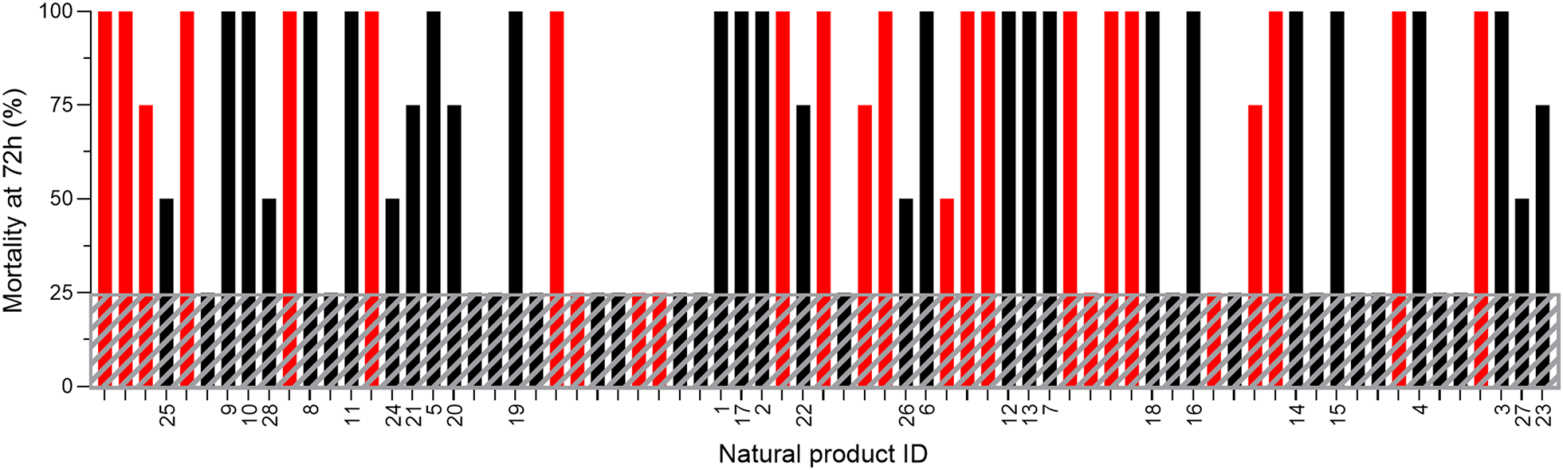
A screening campaign to identify natural products (NPs) with *Aedes aegypti* larvicidal activity. Each bar in the graph represents one of the screened NPs that displayed larvicidal activity; all bars above the grayed-out area represent NPs causing ≥ 50% mortality within 72 h; the red bars represent hazardous NPs that were eliminated from further characterization. NPs were defined as hits if they killed ≥ 50% of the larvae at 50 µM within 72 h and had not been classified as hazardous

In the initial screen for larvicidal activity, four second-instar *Ae. aegypti* larvae were exposed to 50 µM of each natural product (NP) in the breeding water, and survival was then monitored for 72 h. The tested dose of 50 µM was selected based on mainly practical reasons such as available material and dilution volumes in DMSO. Of the 390 compounds tested, 71 caused mortality within 72 h (indicated by bars in Fig. 1). NP hits with known hazardous properties, such as reproductive toxins or compounds with carcinogenic, toxic, highly toxic or poisonous activity, were eliminated from further characterization (red bars in Fig. 1). Setting the mortality rate cutoff at 50% (i.e., at least two of four exposed larvae dying within the 72-h period) resulted in a total of 28 NP hits (Table 1). None of the control larvae were affected under identical conditions during the same time period. Full data from the screening campaign of Natural Products Set V collection are available in Additional file 1: Table S1.

**Table 1:**
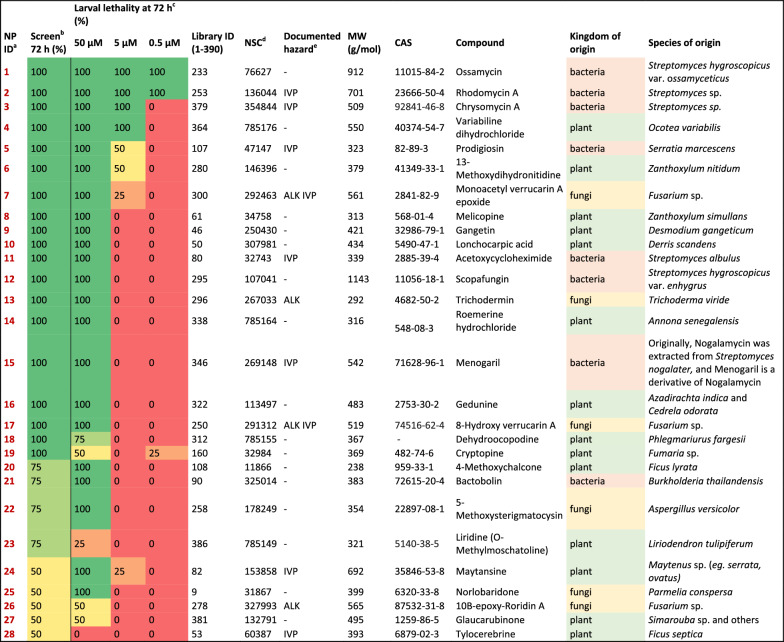
List of larvicidal natural products identified in the screening campaign

^a^Natural product identification number used in this article. ^b^*Ae. aegypti* larval mortality 72 h after NP exposure at 50 µM. ^c^*Ae. aegypti* larval mortality 72 h after NP exposure at 50 µM, 5 µM, and 0.5 µM. ^d^National Service Centre number (a compound identifier assigned by DTP at NCI). ^e^IVP: in vitro/vivo potent, ALK: alkylating agent

### Confirming the larvicidal activity of the 28 NP hits

To confirm and further characterize the larvicidal activity of the 28 NP hits identified in the first screen, L2 *Ae. aegypti* larvae were exposed to the hit NPs at the following three concentrations: 50 µM (as in the screening), 5 µM and 0.5 µM. The assays were performed as described in the Methods section. As many as 26 of the 28 NP hits were confirmed to possess larvicidal activity (Table 1), and the overall hit rate in the library screen was confirmed to be 6.7% (26 out of 390). Only two were identified as false positives and therefore eliminated from further consideration (NP hits 23 and 28). Of the 26 hits, 22 killed all larvae at 50 µM within 72 h, and eight of the hits also displayed larvicidal activity at a 10-fold lower dose (5 µM). Two of the 26 hits (NP hits 1 and 2) killed all larvae at the lowest dose tested (0.5 µM).

Three kingdoms were represented among the larvicidal NPs; plants were the most abundant source (14 hits), followed by bacteria (8 hits) and fungi (6 hits). The molecular weights of the hits ranged from 248 to 1143 g/mol, with a median of 410 g/mol. According to hazard records for these products, nine hits were noted as being in vitro/vivo potent (IVP), four hits were noted as alkylating agents (ALK), and two were noted as both IVP and ALK.

### Screening NP hits for adulticidal activity

**Fig. 2:**
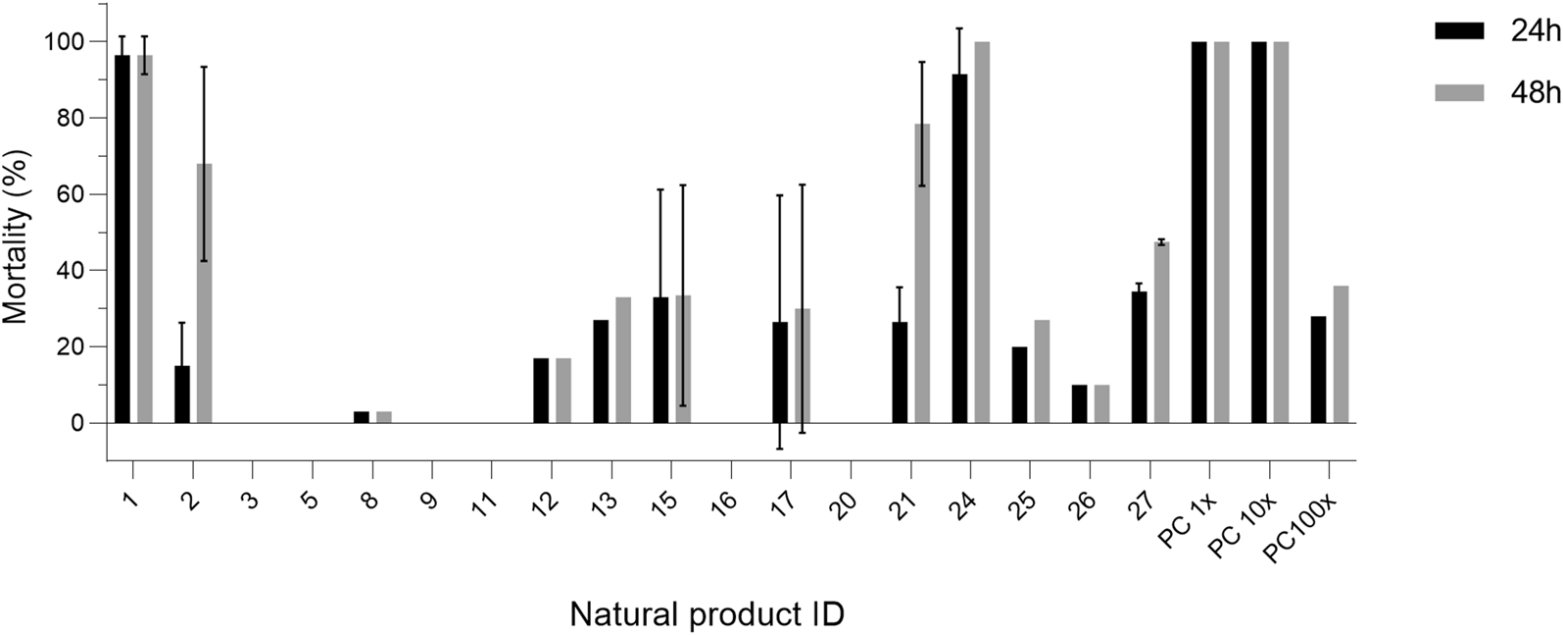
Natural product hits from the larval screen were evaluated for adulticidal activity on *Aedes aegypti* by microinjection into the hemolymph. Bars in the graph represent adult female mosquito mortality at 24 h (black) and 48 h (gray) after injection with 1.4 nmol of one of the 18 hit NPs identified in the screen that was both available and soluble. Thirty mosquitoes were used for each mortality assay, with two independent replicates. Percentages were adjusted according to Abbott’s formula when control mosquito mortality was 5–20%[40]. PC: positive control propoxur in 1× (same as assay), 10× and 100× dilutions

Larger quantities of potential NP hit candidates were needed to be able to investigate whether the confirmed hits also killed adult mosquitoes. Of the 26 confirmed larvicidal NPs, 22 were available from NCI in approximately 5 mg quantities (NP hits 4, 14, 18 and 22 were not available) to enable screening for adulticidal activity. The 22 NP hits were obtained as powders and dissolved in DMSO to a final concentration of 100 mM. Working dilutions of these were prepared in mosquito saline and stored at – 20 °C. Some solubility issues were encountered, and this problem in combination with the small mass of material being used (~ 5 mg) led to an additional four NPs being eliminated from further study (NP hits 6, 7, 10 and 19). To ensure that a consistent and comparable amount of each hit was tested for adulticidal activity, and to consider any chemical properties that might affect a candidate’s ability to cross the insect cuticle, the NPs were directly injected into the thorax of female mosquitoes. Four hits (NP hits 1, 2, 21 and 24), including the two most promising NPs from the larvicidal assays (NP hits 1 and 2), caused > 60% mortality at 48 h after injection of 1.4 nmol NP (Fig. 2). NP hits 1, 2, 21 and 24 killed 97%, 68%, 79% and 100%, respectively, of the adult *Aedes* mosquitoes at this dose. The most potent NP hits, 1 and 24, already caused 97% and 92% mortality, respectively, at 24 h after injection.

These four adulticidal hits were also evaluated at a tenfold lower dose (0.14 nmol). The only NP that retained its full potency at this dose was NP hit 1 (100% mortality). We further confirmed that this NP hit had a similar adulticidal activity as the commercially used carbamate insecticide propoxur (positive control), which also caused 100% mortality at 1.4 and 0.14 nmol, and 36% mortality at 0.014 nmol (Fig. 2). NP hit 1, however, was not tested at this lowest dose. None of the other three NPs displayed adulticidal activity > 25% when tested at 0.14 nmol; NP hit 2 killed 3%, 21 killed 10%, and 24 killed 23% (Additional file 1, Table S2). These results indicate that some NP hits identified in the screen displayed the desirable property of both larvicidal and adulticidal activity.

### Selection of top hits for further characterization

When we combined the results from the larvicidal and adulticidal assays and considered the origin of the products (e.g., availability, producibility), NP hits 1, 21 and 24 were considered the most promising candidates and were therefore selected for further experiments. Most important in the selection criteria was the NP’s ability to kill both adult and larval stages.

NP hit 1 is ossamycin, a natural product isolated from cultures of *S. hygroscopicus* var. *ossamyceticus*. It was first described in 1965 with cytotoxic and antifungal properties [41]. Ossamycin is a member of the macrocyclic polyketide family and, like other members of this family, it has been shown to inhibit electron transport by targeting the F0 component of mitochondrial F1F0-ATPase [42, 43]. More recently, it has been described as selectively cytotoxic towards a number of tumor cell lines, [44], making it interesting as a potential chemotherapeutic agent in cancer therapy [45]. The three-dimensional structure of ossamycin was determined in 1995 [46], and its biosynthetic pathway was elucidated in 2019 [38]. To the best of our knowledge, ossamycin has not previously been explored for insecticidal activity.

NP hit 21 is bactobolin, a natural product isolated from cultures of gram-negative *B. thailandensis*. Its quorum sensing-regulated antibiotic properties were elucidated in 2009 [47], and the responsible molecule was isolated, purified and characterized the year after [48]. As knowledge about the molecule increased, its production was improved and simplified [39], and eventually the structure was determined by NMR; it turned out to be identical to the molecule produced by *Pseudomonas* that already had been described in 1979 [49]. Bactobolin is a member of the polyketide-peptide family and targets ribosomes, thereby blocking protein synthesis by inhibiting translation termination [50, 51].

NP hit 24 is maytansine, a natural product first isolated in 1972 from the Ethiopian shrub *Maytenus serrata* (family Celastraceae) [52]. Maytansine inhibits microtubule assembly, inducing microtubule disassembly and thereby disrupting mitosis by binding to tubulin at the rhizoxin binding site [53]. Maytansine exhibits cytotoxicity against many tumor cell lines and may inhibit tumor growth in vivo [52]. NCI had already initiated clinical trials with maytansine in 1975, but they were unfortunately unfruitful [54]. More recently, maytansine and analogs thereof have been back in focus, now as antibody-targeted conjugates, and in 2013 one analog was approved for clinical use against breast cancer [55].

### Exploring larvicidal and adulticidal activity of selected NPs against *Anopheles gambiae*

**Fig. 3:**
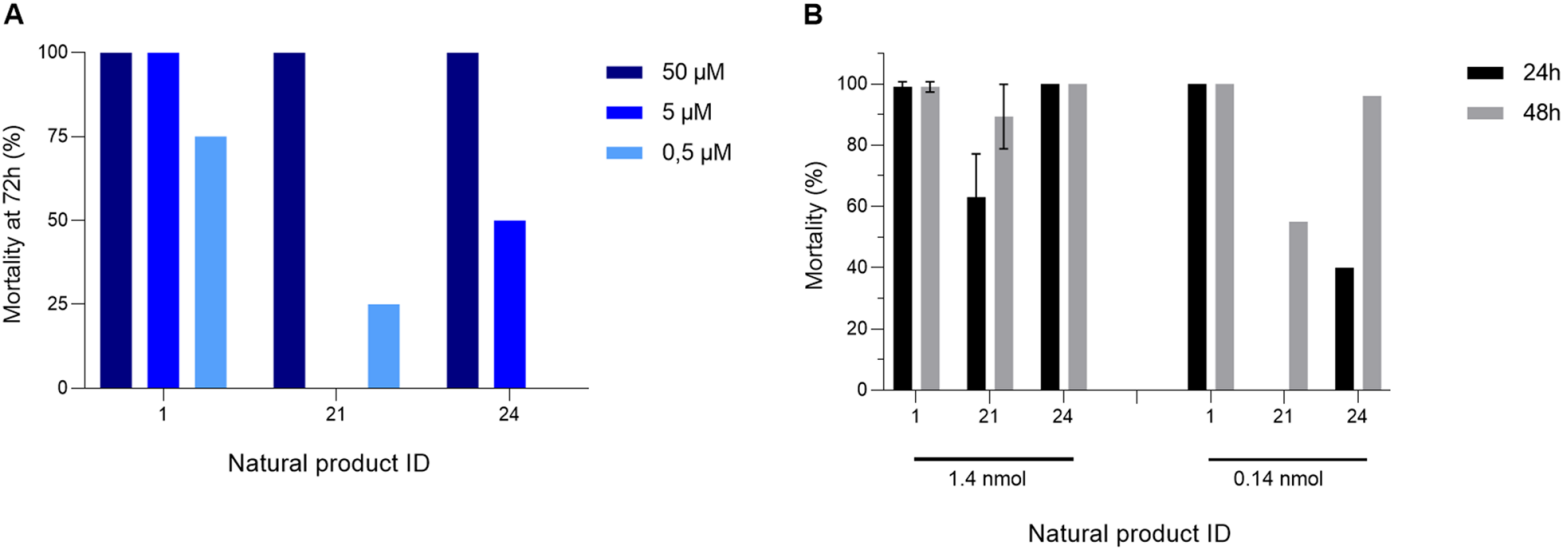
Mosquitocidal activity on *Anopheles gambiae* with natural product hits from the screen against *Aedes aegypti*. **A** Larval mortality 72 h after exposure to NP hits 1, 21 or 24 at three different concentrations (50, 5 and 0.5 µM). **B** Adult mortality 24 h (black bars) and 48 h (gray bars) after injection with 1.4 nmol (left) or 0.14 nmol (right) of NP hits 1, 21 or 24. Thirty mosquitoes were used for each NP, and experiments were run in triplicate (high dose) or single replicate (low dose). Percentages were adjusted according to Abbott’s formula when control mosquito mortality was 5–20% [40]

The three most promising NP hits selected, 1, 21 and 24, were then further assessed for potential *An. gambiae* larvicidal and adulticidal activity. All three NPs displayed *An. gambiae* larvicidal activity (Fig. 3a). NP hits 1, 21 and 24 killed 100% of the larvae at 50 µM within 72 h. NP hit 1 had the strongest potency, with 75% mortality observed at 0.5 µM, the lowest concentration tested. These results were in strong agreement with the larvicidal effect observed for *Aedes*.

Similar to the results for *Aedes*, NP hits 1, 21 and 24 also showed *An. gambiae* adulticidal activity following microinjection into the hemolymph. NP hits 1 and 24 killed 99% and 100% of the mosquitoes within 48 h after injection, and NP hit 21 killed 89% (Fig. 3b). At a tenfold lower dose, NP hit 1 killed 100% of the *Anopheles* mosquitoes, making it the most potent NP in all our experiments. NP hit 24 killed 96%, and NP hit 21 killed 55% of exposed mosquitoes within 48 h after injection at 0.14 nmol (Fig. 3b).

### Testing killing potency through NP ingestion

**Fig. 4:**
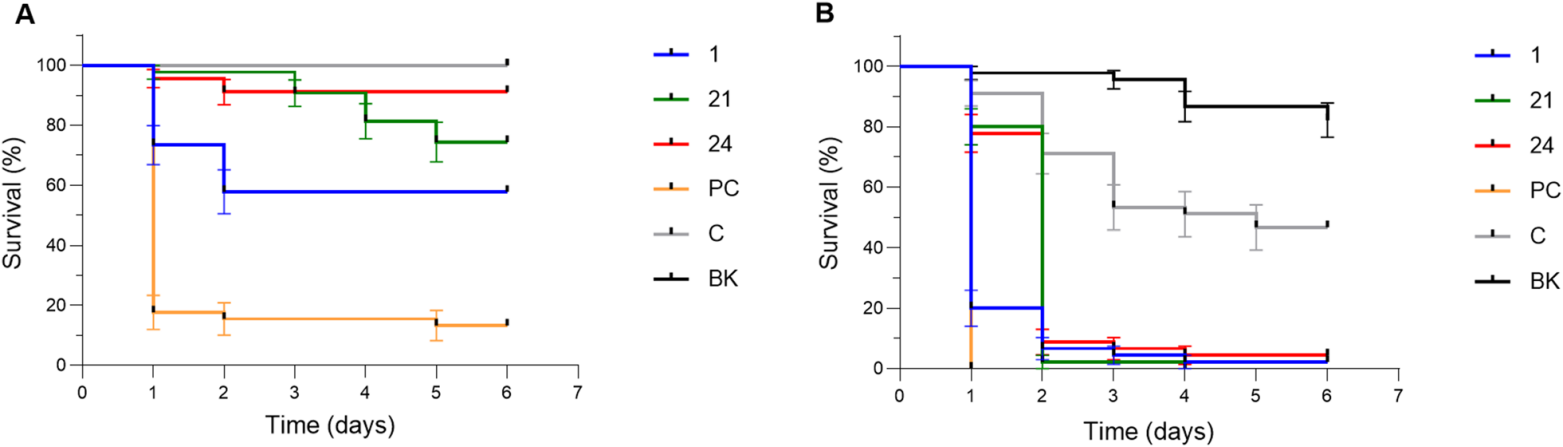
Adulticidal activity on *Aedes aegypti* and *Anopheles gambiae* by feeding different natural product hits. Survival curves for *Ae. aegypti *(**A**) and *An. gambiae* (**B**) adult female mosquitoes after feeding on sugar solution containing 4 mM of NP hit 1 (blue line), 21 (green line), 24 (red line), the positive control (PC) propoxur (yellow line), the control (**C**) treated with solvent DMSO (gray line) or the blank (BK) without any additives (black line)

One delivery route for biopesticides targeting adult mosquitoes is through feeding, and therefore we offered adult female *Aedes* and *Anopheles* mosquitoes a 10% sucrose solution spiked with one of the selected NPs at a final concentration of 4 mM for 24 h. The feeding solution was then changed to sucrose alone, and mosquito survival was monitored daily. NP hit 1 again proved to be the most potent NP in this experiment, killing 43% of the *Aedes* mosquitoes within 2 days (Fig. 4a) and 96% of the *Anopheles* population within 3 days after exposure (Fig. 4b). At the only dose tested, NP hit 21 killed 26% and 24 killed 9% of the *Aedes* mosquitoes within 5 days (Fig. 4a). *Anopheles* mosquitoes were in general more sensitive to all three tested NPs, and mortality was always close to 100% within 5 days after exposure (Fig. 4b). It is possible that the strong mosquitocidal effect seen on *Anopheles* can be attributed in part to the presence of DMSO; however, the mortality was still significantly higher in the NP-fed than in the DMSO-spiked sucrose-fed control mosquitoes.

### Mosquitocidal activity of culture extracts of the source organisms of NP hits 1 (*S. hygroscopicus*) and 21 (*B. thailandensis*)

We have confirmed potent mosquitocidal activities for several purified NPs, suggesting that they could be developed into novel biopesticides for mosquito control. However, large-scale production and purification of the NPs may turn out to be prohibitive from a cost and commercialization standpoint, whereas the preparation of extracts that contain the active NPs from their source organisms can potentially overcome this hurdle. Therefore, we investigated crude extracts of the two bacteria-derived NPs hits, 1 and 21, for mosquitocidal activity. Crude ethyl acetate extractions of bacterial cultures of *S. hygroscopicus* var. *ossamyceticus* (the origin of NP hit 1) and *B. thailandensis* (the origin of NP hit 21) were prepared and added to *Ae. aegypti* larvae breeding water to investigate their potential larvicidal activity. Bacterial culturing and extraction methods were modified from [38] and [39]. Doses of 0.2, 2 and 20 µl of each extract per milliliter of breeding water were used, and larval survival was monitored for 5 days. Control larvae were exposed to extracts of non-bacteria-containing culture medium.

**Fig. 5:**
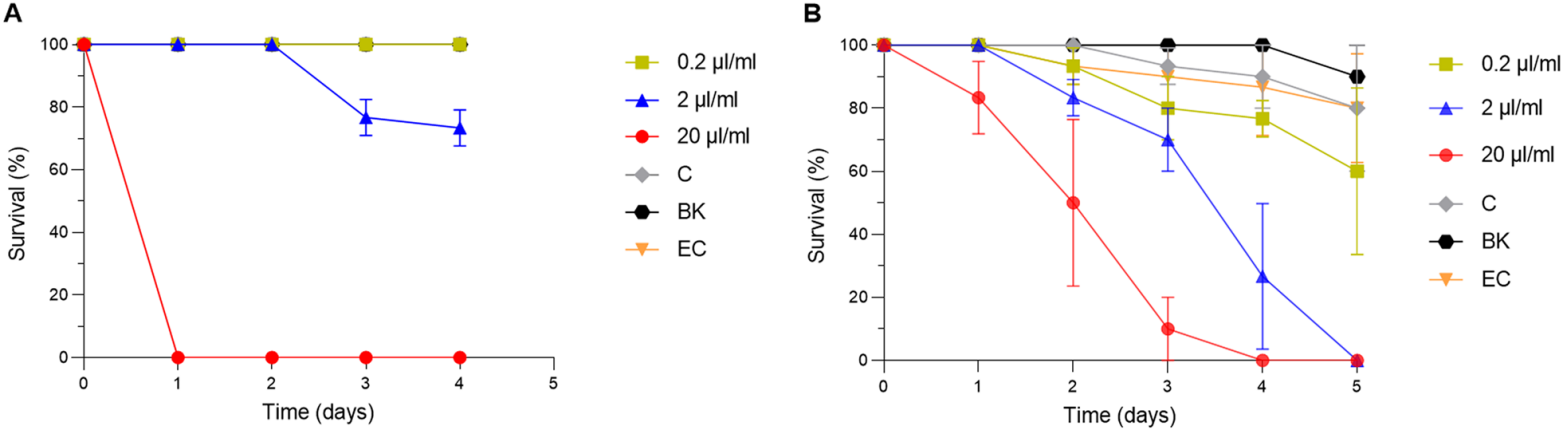
Larvicidal activity of crude extracts on *Aedes aegypti*. Survival curves of *Ae. aegypti* larvae after being exposed to crude extracts of **A** ossamycin from *S. hygroscopicus* cultures or **B** bactobolin from *B. thailandensis* cultures. Larval mortality after exposure to 0.2 μl/ml (green line), 2 μl/ml (blue line) or μl/ml (red line) bacterial extract, or to the control (C) treated with solvent DMSO (gray line), the blank (BK) without any additives (black line), or the extraction control (EC); 20 μl/ml of the product obtained by the extraction with no bacteria (yellow line). Survival is given as the average of three technical replicates

The extracted material from *S. hygroscopicus*, presumably containing the active ingredient ossamycin (NP hit 1), killed 100% of the *Ae. aegypti* larvae within 1 day at the highest dose (20 µl/ml), whereas the 2 µl/ml dose killed approximately 27% within 4 days, and the 0.2 µl/ml dose had no effect (Fig. 5a). Neither the control extract nor the DMSO control caused any mortality.

The extracted material from *B. thailandensis*, presumably containing the active ingredient bactobolin (hit 21), displayed a potent larvicidal effect, with the 20 µl/ml and 2 µl/ml doses killing 100% of the larvae within 4 and 5 days, respectively (Fig. 5b). Even the lowest dose of 0.2 µl/ml killed approximately 30% of the larvae within 5 days. No effect was observed for the control extract or DMSO control.

We also tested *B. thailandensis* extracts for adulticidal activity by allowing mosquitoes to feed on an extract-laced sucrose solution, but observed no significant effect on survival of either *Ae. aegypti* or *An. gambiae* females (Additional file 1: Fig. S1). Further testing is needed to explore possible adulticidal effects as well as larvicidal effects of both extracts on *An. gambiae*.

## Discussion

Screening libraries of known NPs represents a potentially cost-effective approach to identifying new environmentally friendly biopesticides for mosquito control and has mostly been applied for non-mosquito pests [56, 57]. The re-purposing of NPs currently being used or developed for other applications is being considered by the WHO in the search for new much-needed vector-control tools. In this study, we screened a library of 390 NPs (NCI open repository NP library) that had previously been tested for various other activities and properties. To the best of our knowledge, this particular library of natural products has not been previously explored for biological activity against disease-transmitting mosquitoes. The aim of our screening campaign was to identify NPs that could represent new active ingredients for the development of novel biopesticides.

In the screening we found a significant number of hits with promising larvicidal activity on the main arboviral vector *Ae. aegypti*. Since we were using a library of more or less well-studied NPs, we could at this stage already filter out hits with hazardous properties that most likely make them unsuitable for our purposes. This filtering narrowed down the number of hits to a feasible 28, and interestingly four of them turned out to possess both larvicidal and adulticidal effects. Killing activity against multiple mosquito life stages is desirable because it increases a biopesticide’s potential and utility.

The top three NPs that were selected for further characterization were active against the two most important mosquito vectors, *An. gambiae* and *Ae. aegypti*. Importantly, exposure through both direct injections into the hemolymph and oral intake of NPs had adulticidal effect. These results indicate that, at the doses tested, the NPs were not de-activated by digestion after their intake, and they were most likely absorbed by the mosquitoes’ gut epithelium. This property can potentially enable multiple means of exposure, through either attractive toxic sugar baits or contact with the cuticle through spraying, although uptake through the cuticle would have to be enabled by using appropriate adjuvants.

Although the most common insecticide resistance mechanisms identified in the field today are target-site mutations and metabolic resistance [58, 59], the NPs identified as most effective in this study most likely kill mosquitoes through modes of action that differ from those of current chemical insecticides, meaning they do not primarily target acetylcholinesterase or voltage-gated ion channels, where target-site mutations cause problem for current chemical insecticides. Therefore, we hypothesize that NP1, NP21 and NP24 are also potent against mosquito strains that are resistant because of target-site mutations. It will be very interesting and important to evaluate the efficacy of these NPs on both target site-resistant and metabolically resistant mosquito populations in future studies.

The idea is also that crude bacterial extracts are less likely to trigger the evolution of mosquito resistance compared to conventional synthetic insecticides because they likely contain multiple molecules with insecticidal activity. Irrespective of the origin of a product, a single compound is likely to eventually lead to development of resistance. However, for example, Bti-based insecticides consist of a mix of multiple toxins, and thus it is more complex to develop resistance to Bti-preparations and so far no Bti-resistance has been identified in the field [60]. Similarly, a crude bacterial extraction from B. thailandensis or *S. hygroscopicus* might be less likely to cause a rapid resistance development since there are probably several active ingredients in their respective preparations. The tradeoffs between the use of whole organisms, extracts and pure NPs in terms of production complexity, cost, potency and the regulatory pathway leading to approval for use are all important factors to consider in the development of new biopesticides [32, 61]. The fact that crude bacterial extracts of two of the three top NPs identified here retained mosquitocidal activity is promising for future development and commercialization. However, further research is needed to address additional properties of these NPs and their extracts regarding toxicity, shelf life and non-target effects. It will also be necessary to test selected NPs for activity against additional disease vector insects to realize their full potential, including insecticide resistant strains. None of the three most promising hits has, to the best of our knowledge, been reported as insecticidal for mosquitoes, but because of their known in vitro/in vivo potency on medicinally important targets, relevant properties of the pure active molecules are available. The acute toxicity for mice (LD_50_) is 1.8 mg/kg [41], 6.25 mg/kg [62] and 0.25 mg/kg [63] by the intraperitoneal route and 1.8 mg/kg [41], 10 mg/kg [64] and 1.53 mg/kg [65] by the intravenous route for ossamycin, bactobolin and maytansine, respectively. All three NPs thus display a stronger acute toxicity compared to the chemical insecticide propoxur with an LD_50_ of 12 mg/kg [66] intraperitoneally on mice. Concerning shelf life, for the pure compounds all hits appear to be long-lasting: all three are commercially available as powders, and sales companies indicate that they are stable for years under correct storage. One study concludes that bactobolin retained its antimicrobial activity against *E. coli* in filtered culture fluid that had been incubated at 60 °C for 2 h [50]. Accelerated shelf-life assays are planned for the current bacterial extractions to test for product stability and lasting activity. A maytansine analog has been approved for clinical use [55], and thus unwanted non-target effects on humans caused by this product are unlikely; less is known on possible off-target toxicity for the other molecules.

## Conclusions

Vector control is the most widely used method to control mosquito-borne diseases and largely involves synthetic insecticides that may negatively impact the environment and become inefficient because of the emergence of insecticide resistance. Therefore, biopesticides representing natural organisms or substances with mosquito lifespan-shortening effects are receiving increasing attention. We screened 390 natural products to identify 26 molecules that kill the larval stages of the yellow fever mosquito *Aedes aegypti*. Further screening and evaluation showed that a number of natural products also killed the larval stages of the malaria-transmitting mosquito *An. gambiae* as well as the adult form of both species. We also show that crude extracts and preparations from some of the best candidates’ sources (organisms of origin) exert mosquitocidal activity.

## Supplementary Information

**Additional file 1: Table S1.** Full result from the screening campaign of Natural Products Set V collection on *Ae. aegypti* larvae. **Table S2.** Adulticidal activity on *Ae. aegypti* by injections. **Figure S1.** Adulticidal activity of crude extracts on *An. gambiae*. Survival curves of adult *An. gambiae* females after being allowed to feed on an extract-laced sucrose solution containing crude extracts of bactobolin from *B. thailandensis*. Survival is given as the average of three technical replicates.

## Data Availability

All data are available in the main text or the supplementary materials.
