# Supplementary material for: Discovery of novel natural products for mosquito control
Source: Parasit Vectors. 2022 Dec 21;15:481. doi: 10.1186/s13071-022-05594-z (PMC9768913; doi:10.1186/s13071-022-05594-z)
Supplement: Supplementary file 1 — Additional file 1: Table S1. Full result from the screening campaign of Natural Products Set V collection on Ae. aegypti larvae. Table S2. Adulticidal activity on Ae. aegypti by injections. Figure S1. Adulticidal activity of crude extracts on An. gambiae. Survival curves of adult An. gambiae females after being allowed to feed on an extract-laced sucrose solution containing crude extracts of bactobolin from B. thailandensis. Survival is given as the average of three technical replicates. [file 13071_2022_5594_MOESM1_ESM.docx]

**Table S1. Full result from the screening campaign of Natural Products Set V collection on *Ae. aegypti* larvae.**

| NP ID of hits^a^ | Nr (1-390) | Larval lethality at 72h (%)^b^ | NSC^c^ | MW (g/mol) | Documented hazard^d^ |
| --- | --- | --- | --- | --- | --- |
|  | 1 | 100 | 757 | 399 | HTX IRT IVP RPT |
|  | 2 | 0 | 145118 | 460 |  |
|  | 3 | 100 | 7524 | 674 | HTX IRT RPT |
|  | 4 | 0 | 177406 | 591 |  |
|  | 5 | 0 | 12097 | 213 | IRT |
|  | 6 | 0 | 250429 | 364 |  |
|  | 7 | 75 | 26258 | 394 | IRT RPT TOX |
|  | 8 | 0 | 284437 | 248 | TOX |
| 1 | 9 | 50 | 31867 | 398.45 |  |
|  | 10 | 0 | 305222 | 494 | HTX RPT |
|  | 11 | 0 | 36398 | 304.26 |  |
|  | 12 | 0 | 332876 | 318.46 |  |
|  | 13 | 0 | 58368 | 640 | IVP |
|  | 14 | 0 | 361902 | 264 |  |
|  | 15 | 0 | 72116 | 354.45 |  |
|  | 16 | 0 | 401005 | 354 |  |
|  | 17 | 0 | 89671 | 280.36 | IVP TOX |
|  | 18 | 0 | 2080 | 504 |  |
|  | 19 | 100 | 122023 | 1111 | HTX IRT IVP |
|  | 20 | 0 | 3590 | 513.52 |  |
|  | 21 | 0 | 34552 | 185 |  |
|  | 22 | 0 | 5897 | 322 |  |
|  | 23 | 0 | 36407 | 414 |  |
|  | 24 | 0 | 7533 | 985 |  |
|  | 25 | 0 | 43338 | 164 |  |
|  | 26 | 0 | 8661 | 286 |  |
|  | 27 | 0 | 46709 | 131 |  |
|  | 28 | 0 | 9699 | 270 | CRC |
|  | 29 | 0 | 51351 | 284 |  |
|  | 30 | 0 | 13123 | 113 |  |
|  | 31 | 0 | 63946 | 858 |  |
|  | 32 | 0 | 15624 | 382 |  |
|  | 33 | 0 | 72861 | 305 |  |
|  | 34 | 0 | 19509 | 390.91 | IVP TOX |
|  | 35 | 0 | 79404 | 380 |  |
|  | 36 | 0 | 22939 | 224 |  |
|  | 37 | 0 | 85998 | 265.22 | CRC RPT |
|  | 38 | 0 | 26254 | 194 |  |
|  | 39 | 0 | 93373 | 420 |  |
|  | 40 | 0 | 31754 | 256 |  |
|  | 41 | 0 | 2952 | 300 | IRT IVP TOX |
|  | 42 | 25 | 150817 | 926.11 |  |
|  | 43 | 0 | 7668 | 304 |  |
|  | 44 | 0 | 177858 | 823.9 |  |
|  | 45 | 0 | 13252 | 515 | IRT RPT |
| 2 | 46 | 100 | 250430 | 420.5 |  |
|  | 47 | 0 | 26271 | 261.09 | ALK CRC IVP RPT TOX |
|  | 48 | 0 | 285116 | 1648.84 | PSN |
|  | 49 | 0 | 32192 | 367 |  |
| 3 | 50 | 100 | 307981 | 434 |  |
|  | 51 | 0 | 42038 | 261 |  |
|  | 52 | 0 | 333856 | 1336.48 |  |
| 4 | 53 | 50 | 60387 | 393 | IVP |
|  | 54 | 0 | 369397 | 282 |  |
|  | 55 | 0 | 76022 | 429.43 |  |
|  | 56 | 0 | 407286 | 302 | IRT RPT |
|  | 57 | 100 | 94600 | 348 | IVP TOX |
|  | 58 | 0 | 2150 | 152 |  |
|  | 59 | 0 | 122224 | 639 |  |
|  | 60 | 0 | 3716 | 116 |  |
| 5 | 61 | 100 | 34758 | 313 |  |
|  | 62 | 0 | 6435 | 302 |  |
|  | 63 | 0 | 36437 | 376 |  |
|  | 64 | 0 | 7535 | 985 |  |
|  | 65 | 0 | 43339 | 232 |  |
|  | 66 | 0 | 8751 | 86 | COR |
|  | 67 | 0 | 46728 | 376 |  |
|  | 68 | 25 | 10105 | 355 |  |
|  | 69 | 0 | 56410 | 348.36 |  |
|  | 70 | 0 | 14135 | 248 |  |
|  | 71 | 0 | 67392 | 152 |  |
|  | 72 | 0 | 16631 | 154 | IRT |
|  | 73 | 0 | 72862 | 364 |  |
|  | 74 | 0 | 19990 | 770 |  |
|  | 75 | 0 | 81463 | 400 |  |
|  | 76 | 0 | 23615 | 228.2 |  |
|  | 77 | 0 | 86005 | 585.56 |  |
|  | 78 | 0 | 26327 | 242 |  |
|  | 79 | 0 | 93674 | 594.71 |  |
| 6 | 80 | 100 | 32743 | 339 | IVP |
|  | 81 | 100 | 3053 | 1255.43 | HTX IVP RPT |
| 7 | 82 | 50 | 153858 | 692 | IVP |
|  | 83 | 0 | 8519 | 260 | IRT RPT TOX |
|  | 84 | 0 | 180515 | 196 |  |
|  | 85 | 0 | 14975 | 497 |  |
|  | 86 | 0 | 255109 | 546 | ALK IVP |
|  | 87 | 0 | 26326 | 242.27 |  |
|  | 88 | 0 | 287088 | 511 | PSN |
|  | 89 | 0 | 32979 | 341 |  |
| 8 | 90 | 75 | 325014 | 383 |  |
|  | 91 | 0 | 45383 | 506.47 | HTX IVP RPT |
|  | 92 | 0 | 337783 | 362 |  |
|  | 93 | 0 | 62709 | 546 |  |
|  | 94 | 0 | 375294 | 234 |  |
|  | 95 | 0 | 82151 | 564 | CRC IVP TOX |
|  | 96 | 0 | 407306 | 354.45 |  |
|  | 97 | 0 | 96911 | 242 |  |
|  | 98 | 0 | 2347 | 176 |  |
|  | 99 | 0 | 122750 | 561 | ALK IVP |
|  | 100 | 0 | 4143 | 433 |  |
|  | 101 | 0 | 35550 | 431 |  |
|  | 102 | 0 | 6832 | 150 |  |
|  | 103 | 0 | 36508 | 471 |  |
|  | 104 | 0 | 7606 | 158 | TOX |
|  | 105 | 0 | 43871 | 141 |  |
|  | 106 | 0 | 8797 | 393 |  |
| 9 | 107 | 100 | 47147 | 323.44 | IVP |
| 10 | 108 | 75 | 11866 | 238 |  |
|  | 109 | 0 | 59258 | 192 |  |
|  | 110 | 0 | 14664 | 152 |  |
|  | 111 | 25 | 69187 | 276 |  |
|  | 112 | 0 | 18334 | 828 | IVP TOX |
|  | 113 | 0 | 72917 | 396 |  |
|  | 114 | 0 | 20103 | 170 | IRT |
|  | 115 | 0 | 83433 | 328 |  |
|  | 116 | 0 | 23878 | 276 |  |
|  | 117 | 0 | 88466 | 306 |  |
|  | 118 | 0 | 27425 | 175 |  |
|  | 119 | 0 | 95099 | 781 |  |
|  | 120 | 0 | 32944 | 537.53 | HTX IVP |
|  | 121 | 0 | 5113 | 420.46 |  |
|  | 122 | 0 | 157035 | 248 |  |
|  | 123 | 0 | 9665 | 282 |  |
|  | 124 | 0 | 209870 | 547 |  |
|  | 125 | 0 | 15780 | 457 | TOX |
|  | 126 | 0 | 263164 | 314 |  |
|  | 127 | 0 | 29854 | 637 |  |
|  | 128 | 0 | 292567 | 748 | IRT RPT TOX |
|  | 129 | 0 | 32982 | 368.39 | IRT |
|  | 130 | 0 | 325319 | 1112 | HTX IVP |
|  | 131 | 0 | 45923 | 216.19 | CRC TOX |
|  | 132 | 0 | 345647 | 546.53 |  |
|  | 133 | 0 | 63701 | 291 | IVP |
|  | 134 | 0 | 376248 | 475 |  |
|  | 135 | 0 | 85236 | 262.3 | TOX |
|  | 136 | 0 | 614552 | 189 |  |
|  | 137 | 0 | 105388 | 671 |  |
|  | 138 | 0 | 2802 | 186 | IVP |
|  | 139 | 0 | 122819 | 656.66 | CRC IVP |
|  | 140 | 0 | 4586 | 356 |  |
|  | 141 | 0 | 35676 | 220.18 | IRT |
|  | 142 | 0 | 7521 | 530.66 | HTX IVP |
|  | 143 | 0 | 36693 | 332.44 |  |
|  | 144 | 0 | 7616 | 174.11 |  |
|  | 145 | 25 | 44138 | 260 |  |
|  | 146 | 0 | 8973 | 170 |  |
|  | 147 | 0 | 50131 | 464 |  |
|  | 148 | 0 | 11905 | 242 |  |
|  | 149 | 0 | 59263 | 322 | RPT |
|  | 150 | 0 | 14665 | 136 |  |
|  | 151 | 0 | 70931 | 451 | TOX |
|  | 152 | 0 | 18805 | 164 |  |
|  | 153 | 0 | 72942 | 295 |  |
|  | 154 | 0 | 20264 | 347.22 |  |
|  | 155 | 0 | 83436 | 344 |  |
|  | 156 | 0 | 24819 | 414 | IVP TOX |
|  | 157 | 0 | 89937 | 299 |  |
|  | 158 | 0 | 28841 | 196.19 |  |
|  | 159 | 0 | 96021 | 455.68 |  |
| 11 | 160 | 100 | 32984 | 369 |  |
|  | 161 | 0 | 5159 | 641 |  |
|  | 162 | 0 | 169627 | 768 |  |
|  | 163 | 0 | 11440 | 390 |  |
|  | 164 | 0 | 210236 | 334 |  |
|  | 165 | 0 | 22070 | 306 |  |
|  | 166 | 0 | 270914 | 447 |  |
|  | 167 | 0 | 30552 | 410 | TOX |
|  | 168 | 0 | 301683 | 262 |  |
|  | 169 | 25 | 35611 | 905 |  |
|  | 170 | 0 | 330753 | 587 |  |
|  | 171 | 0 | 51001 | 842 |  |
|  | 172 | 0 | 349438 | 168.19 | HTX |
|  | 173 | 100 | 67574 | 923.04 | HTX IRT IVP RPT |
|  | 174 | 0 | 382796 | 551.55 |  |
|  | 175 | 0 | 85239 | 262 |  |
|  | 176 | 0 | 824 | 200 |  |
|  | 177 | 0 | 114344 | 276 |  |
|  | 178 | 0 | 2835 | 321 |  |
|  | 179 | 0 | 129536 | 310 | ALK |
|  | 180 | 0 | 5379 | 271 |  |
|  | 181 | 0 | 36294 | 248 |  |
|  | 182 | 0 | 7525 | 693 | IVP TOX |
|  | 183 | 0 | 38010 | 309 |  |
|  | 184 | 0 | 7652 | 267 |  |
|  | 185 | 0 | 44175 | 292 |  |
|  | 186 | 25 | 9170 | 373 | IVP TOX |
|  | 187 | 0 | 50132 | 446 |  |
|  | 188 | 0 | 12444 | 182 |  |
|  | 189 | 0 | 61809 | 398 |  |
|  | 190 | 0 | 14974 | 396 |  |
|  | 191 | 0 | 72138 | 579 |  |
|  | 192 | 25 | 19028 | 222 | IRT |
|  | 193 | 0 | 75527 | 358 |  |
|  | 194 | 0 | 21725 | 244 |  |
|  | 195 | 0 | 83439 | 334 |  |
|  | 196 | 0 | 24872 | 258 |  |
|  | 197 | 25 | 90636 | 907 |  |
|  | 198 | 0 | 30238 | 148 |  |
|  | 199 | 0 | 98542 | 381 | IRT TOX |
|  | 200 | 25 | 33410 | 462 | IVP TOX |
|  | 201 | 0 | 5366 | 413 | PSN TOX |
|  | 202 | 0 | 176503 | 362 |  |
|  | 203 | 25 | 11926 | 341 | CRC RPT TOX |
|  | 204 | 0 | 226080 | 914.18 | IVP |
|  | 205 | 0 | 23969 | 346 |  |
|  | 206 | 0 | 284200 | 345 |  |
|  | 207 | 0 | 31048 | 611 |  |
|  | 208 | 0 | 302289 | 232 |  |
|  | 209 | 0 | 36351 | 339 |  |
|  | 210 | 0 | 332598 | 625.76 | IVP |
|  | 211 | 0 | 56464 | 646 |  |
|  | 212 | 0 | 350085 | 270 |  |
|  | 213 | 0 | 71795 | 246 | TOX |
|  | 214 | 0 | 400978 | 248 | IVP TOX |
|  | 215 | 0 | 87511 | 386.31 |  |
|  | 216 | 0 | 1115 | 192 |  |
|  | 217 | 0 | 118343 | 236 |  |
|  | 218 | 0 | 3071 | 152 |  |
|  | 219 | 0 | 133100 | 721 |  |
|  | 220 | 25 | 5863 | 146 |  |
|  | 221 | 0 | 36354 | 507 | IVP TOX |
|  | 222 | 0 | 7532 | 969 |  |
|  | 223 | 0 | 38270 | 1197 | IVP TOX |
|  | 224 | 0 | 8625 | 174 |  |
|  | 225 | 0 | 45384 | 520.5 |  |
|  | 226 | 0 | 9248 | 194 |  |
|  | 227 | 0 | 50393 | 224 |  |
|  | 228 | 0 | 12865 | 405 |  |
|  | 229 | 0 | 62786 | 183 | ALK TOX |
|  | 230 | 0 | 15307 | 322 |  |
|  | 231 | 25 | 72715 | 605 |  |
|  | 232 | 0 | 19038 | 344 |  |
| 12 | 233 | 100 | 76627 | 912 |  |
|  | 234 | 0 | 21728 | 335.36 |  |
|  | 235 | 0 | 85235 | 246 |  |
|  | 236 | 0 | 24951 | 350 | TOX |
|  | 237 | 0 | 93047 | 229 |  |
|  | 238 | 0 | 30625 | 411 | CRC TOX |
|  | 239 | 0 | 99791 | 334 |  |
|  | 240 | 0 | 34202 | 503 |  |
|  | 241 | 0 | 99792 | 350 |  |
|  | 242 | 0 | 236580 | 594 |  |
|  | 243 | 0 | 100858 | 379 |  |
|  | 244 | 0 | 250682 | 364 | ALK |
|  | 245 | 0 | 106969 | 360 |  |
|  | 246 | 0 | 266071 | 410 |  |
|  | 247 | 0 | 112906 | 182.22 | TOX |
|  | 248 | 0 | 269754 | 549 | IRT IVP TOX |
|  | 249 | 0 | 121859 | 272 |  |
| 13 | 250 | 100 | 291312 | 519 | ALK IVP |
|  | 251 | 0 | 127445 | 240 |  |
|  | 252 | 0 | 316458 | 263.26 | IVP |
| 14 | 253 | 100 | 136044 | 701 | IVP |
|  | 254 | 0 | 332294 | 433 |  |
|  | 255 | 0 | 145612 | 755 |  |
|  | 256 | 100 | 355637 | 429 | IVP TOX |
|  | 257 | 0 | 159632 | 236 |  |
| 15 | 258 | 75 | 178249 | 354 |  |
|  | 259 | 0 | 180516 | 302 |  |
|  | 260 | 0 | 407806 | 374.52 | HTX |
|  | 261 | 0 | 642099 | 406 |  |
|  | 262 | 0 | 785153 | 331.84 |  |
|  | 263 | 0 | 785161 | 339.39 |  |
|  | 264 | 0 | 785169 | 325.4 |  |
|  | 265 | 0 | 785178 | 325.36 |  |
|  | 266 | 0 | 785189 | 422.32 |  |
|  | 267 | 0 | 99794 | 410 |  |
|  | 268 | 0 | 237671 | 1330 |  |
|  | 269 | 100 | 100880 | 389.36 | HTX IVP |
|  | 270 | 0 | 255112 | 673 |  |
|  | 271 | 25 | 106995 | 314.25 |  |
|  | 272 | 0 | 266535 | 307.26 | RPT |
|  | 273 | 0 | 112907 | 248.24 |  |
|  | 274 | 75 | 269756 | 562.61 | IRT IVP TOX |
|  | 275 | 0 | 121860 | 352 |  |
|  | 276 | 100 | 292222 | 635 | IRT TOX |
|  | 277 | 0 | 127473 | 242 |  |
| 16 | 278 | 50 | 327993 | 565 | ALK |
|  | 279 | 0 | 335989 | 439 |  |
| 17 | 280 | 100 | 146396 | 379 |  |
|  | 281 | 0 | 359079 | 570 |  |
|  | 282 | 50 | 165563 | 548.59 | HTX IVP |
|  | 283 | 0 | 256942 | 579.99 | TOX |
|  | 284 | 0 | 184398 | 486 |  |
|  | 285 | 100 | 526417 | 1101 | IVP RPT TOX |
|  | 286 | 0 | 661755 | 877 |  |
|  | 287 | 0 | 785154 | 293.36 |  |
|  | 288 | 0 | 785162 | 339.39 |  |
|  | 289 | 0 | 785170 | 362.29 |  |
|  | 290 | 0 | 785179 | 355.39 |  |
|  | 291 | 0 | 99799 | 364 |  |
|  | 292 | 0 | 244387 | 328 |  |
|  | 293 | 0 | 102816 | 244.21 | CRC TOX |
|  | 294 | 100 | 259968 | 773 | IVP TOX |
| 18 | 295 | 100 | 107041 | 1142.47 |  |
| 19 | 296 | 100 | 267033 | 292 | ALK |
|  | 297 | 0 | 113087 | 341 |  |
|  | 298 | 0 | 269760 | 563 | IRT |
|  | 299 | 0 | 121865 | 412 |  |
| 20 | 300 | 100 | 292463 | 561 | ALK IVP |
|  | 301 | 0 | 128487 | 315 |  |
|  | 302 | 100 | 328166 | 535 | ALK IVP TOX |
|  | 303 | 0 | 141538 | 296 |  |
|  | 304 | 0 | 337851 | 696 |  |
|  | 305 | 0 | 147340 | 302 | IVP |
|  | 306 | 25 | 361792 | 401 | HTX IVP |
|  | 307 | 0 | 169517 | 316 |  |
|  | 308 | 100 | 320301 | 469 | HTX IVP |
|  | 309 | 100 | 186301 | 416 | HTX IVP |
|  | 310 | 0 | 601422 | 523 |  |
|  | 311 | 0 | 719655 | 379 |  |
| 21 | 312 | 100 | 785155 | 367.4 |  |
|  | 313 | 0 | 785163 | 385.41 |  |
|  | 314 | 0 | 785173 | 608.72 |  |
|  | 315 | 0 | 785180 | 338.44 |  |
|  | 316 | 0 | 99804 | 510 |  |
|  | 317 | 25 | 247562 | 428 | IVP |
|  | 318 | 0 | 104943 | 418 |  |
|  | 319 | 0 | 265211 | 846 | IVP TOX |
|  | 320 | 0 | 107453 | 333 |  |
|  | 321 | 0 | 269146 | 1085 | HTX IVP |
| 22 | 322 | 100 | 113497 | 483 |  |
|  | 323 | 0 | 278619 | 256 |  |
|  | 324 | 0 | 123383 | 298 |  |
|  | 325 | 0 | 294408 | 487 |  |
|  | 326 | 0 | 129230 | 349 |  |
|  | 327 | 25 | 328426 | 805 | IVP TOX |
|  | 328 | 0 | 142227 | 326 |  |
|  | 329 | 0 | 343256 | 478 |  |
|  | 330 | 0 | 148790 | 433 |  |
|  | 331 | 25 | 365793 | 248 |  |
|  | 332 | 0 | 170365 | 1664.88 |  |
|  | 333 | 75 | 403148 | 398 | IVP TOX |
|  | 334 | 100 | 208734 | 812 | IVP TOX |
|  | 335 | 0 | 607097 | 357.41 |  |
|  | 336 | 0 | 785145 | 295.38 |  |
|  | 337 | 0 | 785156 | 263.29 |  |
| 23 | 338 | 100 | 785164 | 315.8 |  |
|  | 339 | 0 | 785174 | 622.75 |  |
|  | 340 | 25 | 785182 | 406.28 |  |
|  | 341 | 0 | 99843 | 291 | IVP |
|  | 342 | 0 | 248605 | 715 |  |
|  | 343 | 0 | 105827 | 325.34 |  |
|  | 344 | 0 | 265450 | 729.78 | IVP |
|  | 345 | 0 | 108088 | 354 |  |
| 24 | 346 | 100 | 269148 | 542 | IVP |
|  | 347 | 0 | 114341 | 229 |  |
|  | 348 | 0 | 281245 | 391 |  |
|  | 349 | 0 | 123389 | 325 |  |
|  | 350 | 0 | 295426 | 350 |  |
|  | 351 | 0 | 135962 | 482 | IVP |
|  | 352 | 0 | 330500 | 560.69 |  |
|  | 353 | 0 | 143648 | 346 | HTX IVP |
|  | 354 | 25 | 349155 | 291 |  |
|  | 355 | 25 | 156219 | 786 |  |
|  | 356 | 100 | 376128 | 785.1 | HTX IVP |
|  | 357 | 0 | 172946 | 385 | HTX IVP |
|  | 358 | 0 | 403169 | 321 | IVP TOX |
|  | 359 | 0 | 216128 | 490 | HTX IRT IVP |
|  | 360 | 0 | 616348 | 623.15 | IRT RPT |
|  | 361 | 0 | 785148 | 347.8 |  |
|  | 362 | 0 | 785157 | 309.32 |  |
|  | 363 | 0 | 785166 | 297.35 |  |
| 25 | 364 | 100 | 785176 | 549.54 |  |
|  | 365 | 0 | 785186 | 297.35 |  |
|  | 366 | 25 | 100290 | 606 |  |
|  | 367 | 0 | 248958 | 307 | TOX |
|  | 368 | 0 | 106486 | 268 |  |
|  | 369 | 0 | 266032 | 156 | ALK |
|  | 370 | 25 | 111041 | 189.17 |  |
|  | 371 | 0 | 269753 | 549 | IRT IVP TOX |
|  | 372 | 0 | 121849 | 425 |  |
|  | 373 | 100 | 283445 | 519 | ALK IVP TOX |
|  | 374 | 0 | 123977 | 551 |  |
|  | 375 | 0 | 302979 | 374 |  |
|  | 376 | 0 | 136035 | 221 |  |
|  | 377 | 0 | 330917 | 237 |  |
|  | 378 | 0 | 145150 | 260 |  |
| 26 | 379 | 100 | 354844 | 508.52 | IVP |
|  | 380 | 0 | 156236 | 246 |  |
| 27 | 381 | 50 | 132791 | 495 |  |
|  | 382 | 0 | 179834 | 487 | ALK |
|  | 383 | 0 | 407308 | 448 |  |
|  | 384 | 0 | 218321 | 268.27 | RPT TOX |
|  | 385 | 0 | 637086 | 278 |  |
| 28 | 386 | 75 | 785149 | 321.33 |  |
|  | 387 | 0 | 785160 | 311.33 |  |
|  | 388 | 0 | 785167 | 297.35 |  |
|  | 389 | 0 | 785177 | 317.81 |  |
|  | 390 | 0 | 785188 | 325.36 |  |
|  |  |  |  |  |  |

^a^Natural product identification number used in this article. ^b^*Ae. aegypti* larval mortality 72 h after NP exposure at 50 µM. ^c^National Service Center number (a compound identifier assigned by DTP at NCI). ^e^IVP: *in vitro/vivo* potent, ALK: alkylating agent, HTX: highly toxic, TOX: toxic, IRT: irritant, RPT: reproductive toxin, CRC: carcinogenic, PSN: poisonous.

**Table S2. Adulticidal activity^a^ on *Ae. aegypti* by injections.**

|  | 1.4 nmol | | 0.14 nmol | |
| --- | --- | --- | --- | --- |
| **NP ID** | **24 h** | **48 h** | **24 h** | **48 h** |
| **1** | 97 | 97 | 100 | 100 |
| **2** | 15 | 68 | 13 | 13 |
| **3** | solubility issues | | 17 | 7 |
| **4** | not available for more material | | | |
| **5** | solubility issues | | 3 | 0 |
| **6** | solubility issues | | | |
| **7** | solubility issues | | | |
| **8** | 3 | 3 | - | - |
| **9** | 0 | 0 | - | - |
| **10** | solubility issues | | | |
| **11** | 0 | 0 | - | - |
| **12** | 17 | 17 | - | - |
| **13** | 27 | 33 | -1 | -7 |
| **14** | not available for more material | | | |
| **15** | 33 | 35 | 10 | 10 |
| **16** | 0 | 0 | - | - |
| **17** | 27 | 30 | - | - |
| **18** | not available for more material | | | |
| **19** | solubility issues | | | |
| **20** | 0 | 0 | - | - |
| **21** | 27 | 79 | 10 | 10 |
| **22** | not available for more material | | | |
| **23** | eliminated as false positive | | | |
| **24** | 92 | 100 | 3 | 23 |
| **25** | 20 | 27 | - | - |
| **26** | 10 | 10 | - | - |
| **27** | 36 | 48 | - | - |
| **28** | eliminated as false positive | | | |

^a^Adulticidal activity is given as percent mortality at 24 h and 48 h after injection with 1.4 nmol or 0.14 nmol of one of the 22 hit NPs identified in the screen. Thirty mosquitoes were used for each mortality assay and percentages were adjusted according to Abbott’s formula when control mosquito mortality was 5%-20%.


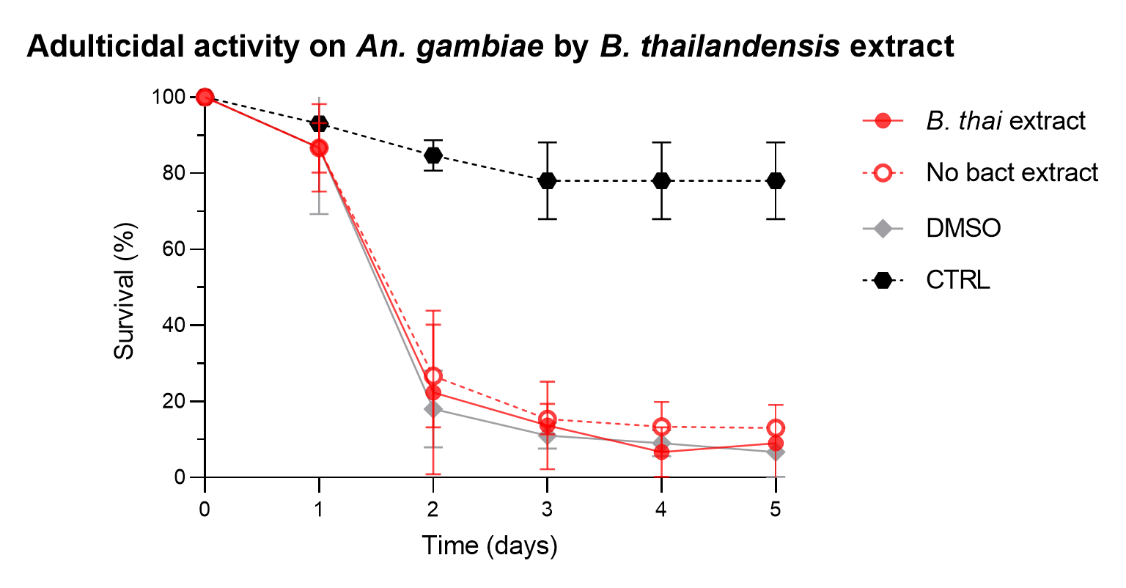


**Figure S1. Adulticidal activity of crude extracts on *An. gambiae*.** Survival curves of adult *An. gambiae* females after being allowed to feed on an extract-laced sucrose solution containing crude extracts of bactobolin from *B. thailandensis.* Survival is given as the average of three technical replicates.
